# What Is the Relationship Between Body Mass Index, Sex Hormones, Leptin, and Irisin in Children and Adolescents? A Path Analysis

**DOI:** 10.3389/fped.2022.823424

**Published:** 2022-04-15

**Authors:** Li Zhang, Xingwang Peng, Yuanyuan Wang, Ruiyao Cao, Zizhe Zhang, Lianguo Fu

**Affiliations:** Department of Child and Adolescent Health, School of Public Health, Bengbu Medical College, Bengbu, China

**Keywords:** children and adolescents, BMI, E2, T, leptin, irisin

## Abstract

**Objective:**

The present research aimed to study the relationship between body mass index (BMI), sex hormones, leptin, and irisin in children and adolescents with different body types.

**Methods:**

In this study, a stratified cluster random sampling method was used to select students aged 8-15 years from two 9-year schools as the research subjects. Based on a case-control study, 183 overweight/obese students were selected. After using sex and age matching to create a matched sample of *normal-weighted* students, a total of 366 students, including 214 boys (58.5%) and 152 girls (41.5%) were included. We measured their height and weight and calculated their body mass index BMI. Afterward, their concentrations of leptin, irisin, oestradiol (E2), and testosterone (T) in the serum were detected.

**Results:**

There were significant differences in T, E2, leptin, and irisin between normal-weighted boys and girls (*p* < 0.05). There were statistically significant differences in T, E2, and irisin between overweight/obese boys and girls (*p* < 0.05). Overweight/obese students had higher concentrations of irisin and leptin than normal-weight students *(p* < 0.05). The direct effect of BMI on irisin was not statistically significant in either normal or overweight/obese students, but their indirect effects *via* leptin were statistically significant (for normal-weight boys and girls, standardized indirect effect coefficient: 0.29 and 0.38, respectively; for overweight/obese boys and girls, standardized indirect effect coefficient: 0.36 and 0.34, respectively). There was a negative pathway of E2 → leptin → irisin in normal-weight boys (standardized indirect effect coefficient: −0.24) and a negative pathway of T → leptin → irisin in overweight/obese boys (standardized indirect effect coefficient: −0.27).

**Conclusion:**

The indirect effects of BMI on irisin *via* leptin exist in children and adolescents of different body types. E2 was negatively correlated with leptin in normal-weight boys, whereas T was negatively correlated with leptin in overweight/obese boys.

## Introduction

In children and adolescents, obesity has become an important public health problem that affects their health into adulthood. It is associated with an increased risk of type 2 diabetes mellitus (T2DM), metabolic syndrome (MS), cardiovascular disease (CVD), and different cancers (1, 2).

Irisin was first discovered by Boström et al. (3) in 2012. It is a factor secreted by muscle and fat and one of the most studied motion-induced peptides in recent years. It acts on both fat and muscle tissue and belongs to the adiponectin group. Serum irisin can reflect the metabolic status (4). In addition, it has been demonstrated that serum-circulating irisin levels are associated with significantly increased fibronectin type III domain protein 5 (FNDC5) gene expression in adipose tissue (5, 6). The expression of FNDC5 in fat is approximately 1/100 of that in muscle (7). The FNDC5 is a widely distributed transmembrane glycoprotein that can be proteolytically cleaved as irisin. The FNDC5 and irisin are reported to play critical roles in regulating inflammation, oxidative stress, autophagy, apoptosis, and pathophysiological processes that cause mitochondrial disruption (8). Irisin is mainly released by white subcutaneous adipose tissue (SAT) (9). It plays a key regulatory role in the conversion of white fat to brown fat, and is a thermogenic protein that promotes energy expenditure through the browning of white fat, suggesting its potential role in inhibiting fat storage and obesity and improving metabolic status (10). Recent reports have indicated that irisin is associated with metabolic disorders, such as obesity (11), insulin resistance (IR) (12), musculoskeletal health (13), type 1 diabetes mellitus (T1DM) (14), and T2DM (15). However, there have been few studies on the mechanism of interaction between BMI and serum irisin concentration in children and adolescents.

Leptin is a product of the obesity gene secreted by fat cells and is thought to play a central role in regulating appetite and energy expenditure. It leads to weight loss by influencing food intake and increasing energy expenditure, and is thought to control food intake at least in part through neuropeptide Y (NPY). Leptin is not only a molecule that reflects energy storage in the body, but is also an important cytokine involved in many physiological functions, such as the inflammatory response, insulin sensitivity, bone metabolism, immunity, and, most importantly, reproductive function (16). High leptin levels are an important manifestation of obesity, and children and adolescents show increased leptin levels with increasing body fat mass (17). Studies have shown that leptin increases the expression of the FNDC5 gene through the activation of nitric oxide synthase (NOS) (18). Leptin also triggers mRNA expression of PRDM16, a protein that regulates muscle fat metabolism and controls the expression of genes specific to the transformation process between skeletal muscle and brown fat cells, which is necessary for browning white fat (19).

The levels of sex hormone secretion in obese children change significantly compared with those in normal children. The testosterone (T) level in obese boys decreased and the oestradiol (E2) level increased, whereas the T and E2 level in obese girls increased (20–22). Studies have found that sex differences in the regulation of leptin synthesis are mediated by steroid hormones (23). Particularly, T inhibits leptin secretion and leptin mRNA production in adipocytes, while E2 positively regulates obese gene expression and leptin secretion in female adipocytes (24). Tanaka et al. (25) showed that E2 increased serum leptin concentrations and leptin mRNA expression in adipose tissue of immature rats. Ulker et al. (26) demonstrated that chronic irisin administration had different effects on pubertal maturation and reproductive system in male and female rats. Luo et al. (27) indicated that the circulating irisin level in serum controls the expression of gonadotropin-releasing hormone (GnRH) in the hypothalamus and regulates the secretion of follicle-stimulating hormone (FSH) and luteinizing hormone (LH). The FSH and LH stimulate the secretion of E2 by follicular granulosa cells and T by Leydig cells, respectively. The E2 can directly induce irisin secretion or act through the anabolic pathway to increase female skeletal muscle mass and promote irisin secretion (28). Irisin levels are higher in obese men than in women, suggesting that the physiological effects of irisin may vary by sex. In animal models, circulating irisin and body weight increased significantly in ovariectomized female rats but not in orchiectomized male rats (29). Therefore, BMI, E2, T, leptin, and irisin may correlate with each other. However, their path associations are still unclear in different body types. The present study aimed to study the relationship between BMI, sex hormones, leptin, and irisin in children and adolescents with different body types. Understanding these issues may help to better clarify the metabolic mechanisms in pediatric obesity.

## Materials and Methods

### Subjects

In this study, a stratified cluster random sampling method was used to select students aged 8-15 years among two 9-year schools as the research subjects. Based on the case-control study, 183 overweight/obese children were selected. After gender and age matching to create a matched sample of children of *normal-weighted*, a total of 366 students, including 214 boys (58.5%) and 152 girls (41.5%), were selected. This study was approved by the Medical Ethics Committee of the Bengbu Medical College [(2015) No. 003] and was conducted in accordance with the Declaration of Helsinki. The participants' guardians signed informed consent forms.

### Morphological Measurement

All morphological variables were measured by staff trained in standardization after children and adolescents had removed their shoes and heavy clothing and were at ease with an empty stomach. Body height was measured to the nearest 0.1 cm using a mechanical scale with a height meter, and a digital body scale was used to measure the body weight to the nearest 0.1 kg.

### Criteria for Overweight/Obese

Body mass index was calculated as weight (kg)/height (m)^2^, and overweight/obese was determined according to the Health industry Standard of the People's Republic of China (WS/T 586-2018). Screening of overweight and obesity in school-age children and adolescents (30).

### Serum Hormone Detection

Venous blood samples of the subjects (~3 ml) after at least 8 h of overnight fasting were collected by medical staff, who had received standardized training, and serum concentrations of irisin and leptin were detected by double-antibody sandwich ELISA. A Human Irisin ELISA Kit and Human Leptin (LEP) ELISA Kit were obtained from Wuhan Huamei Biological Engineering Company. The serum circulating T and E2 levels were assayed using the radioimmunoassay method with a Testosterone Radioimmunoassay Kit and Oestradiol Radioimmunoassay Kit provided by the DIA Company in Shanghai.

### Statistical Analysis

Statistical Package for the Social Sciences (SPSS) 23.0 software was used for the analysis of the data. The abnormal data were analyzed after natural logarithmic transformation to normalize the distribution of all subsequent statistical analyses, including structural equation modeling. The quantitative data were described as the mean ± SD, and a *t*-test was used to investigate the role of E2, T, leptin, and irisin in different body types and their differences between boys and girls. Pearson correlation was used to analyze the correlation between each index. The AMOS 24.0 was used to construct the structural equation model, and the bootstrap program was used to test the indirect effect. The *p* < 0.05 was considered a statistically significant difference.

## Results

### Basic Information

A total of 366 students were enrolled in this study, including 214 boys (58.5%) and 152 girls (41.5%). The concentration of E2, T, leptin, and irisin between boys and girls (overweight/obese and normal weight) was significantly different. However, there were some overweight or obese boys and girls, in whom the leptin concentration was not significantly different. Data are shown in Table 1.

**Table 1:** General situational analysis of research objects (Mean ± SD).

	**Boys**				**Girls**			
**Variables**	**Normal-weighted**	**Overweight/obese**			**Normal-weighted**	**Overweight/obese**		
	**(*n* = 107)**	**(*n* = 107)**	***t***	***P***	**(*n* = 76)**	**(*n* = 76)**	***t***	***P***
Age	10.79 ± 1.63	10.74 ± 1.57	0.256	>0.05	10.63 ± 1.76	10.68 ± 1.70	−0.188	>0.05
LnE2	1.98 ± 1.44	1.93 ± 1.23	0.270	>0.05	2.44 ± 1.65^a^	3.00 ± 1.63^b^	−2.114	<0.05
LnT	3.90 ± 1.66	4.12 ± 1.31	−1.053	>0.05	3.09 ± 1.24^a^	3.41 ± 0.99^b^	−1.729	>0.05
Lnleptin	0.38 ± 1.14	2.31 ± 0.84	−13.979	<0.05	1.40 ± 1.03^a^	2.49 ± 1.20	−5.984	<0.05
Lnirisin	2.13 ± 1.13	3.54 ± 1.30	−8.441	<0.05	3.11 ± 1.12^a^	4.00 ± 1.05^b^	−5.061	<0.05

a *Comparison of hormones in normal-weighted boys and girls P < 0.05;*

b *Comparison of hormones in overweight/obese boys and girls p < 0.05*.

### Univariate Analysis of BMI, T, E2, Leptin, and Irisin in Children and Adolescents With Different Body Types

A slight correlation was found between BMI and E2, T, leptin, and irisin participants of all groups. Leptin and irisin were highly correlated in all groups. A slight correlation was found between BMI and E2, T, leptin, and irisin participants of all groups, with the exception of boys and girls with overweight or obese. Leptin and irisin were highly correlated in all groups. A slight correlation was also found between E2 and leptin in boys and girls with normal-weighted, T and leptin in boys and girls with overweight or obese and between E2 and irisin in girls with normal-weighted. The T was significantly associated with leptin in overweight/obese boys and girls. Data are shown in Table 2.

**Table 2:** Correlation coefficient between body mass index (BMI), testosterone (T), oestradiol (E2), leptin, and irisin in children and adolescents with different body types.

**Gender**	**Variables**	**BMI**	**LnE2**	**LnT**	**Lnleptin**	**Lnirisin**
**Boys**						
**Normal-weighted**
	BMI	1				
	LnE2	0.21^*^	1			
	LnT	0.36^**^	0.24^*^	1		
	Lnleptin	0.39^**^	−0.29^**^	−0.09	1	
	Lnirisin	0.33^**^	−0.16	−0.14	0.60^**^	1
**Overweight/obese**
	BMI	1				
	LnE2	0.12	1			
	LnT	0.34^**^	0.25^*^	1		
	Lnleptin	0.42^**^	−0.19	−0.23^*^	1	
	Lnirisin	0.27^**^	0.1	−0.01	0.64^**^	1
**Girls**						
**Normal-weighted**
	BMI	1				
	LnE2	0.28^**^	1			
	LnT	0.23^**^	0.34^**^	1		
	Lnleptin	0.52^**^	0.23^*^	0.11	1	
	Lnirisin	0.47^**^	0.32^**^	0.15	0.75^**^	1
**Overweight/obese**
	BMI	1				
	LnE2	0.21	1			
	LnT	0.30^**^	0.34^**^	1		
	Lnleptin	0.46^**^	0.05	0.27^*^	1	
	Lnirisin	0.26^*^	0.12	0.14	0.73^**^	1

* *p < 0.05;*

** *p < 0.01*.

### Multifactor Analysis of BMI, T, E2, Leptin, and Irisin in Children and Adolescents With Different Body Types

**Figure 1:**
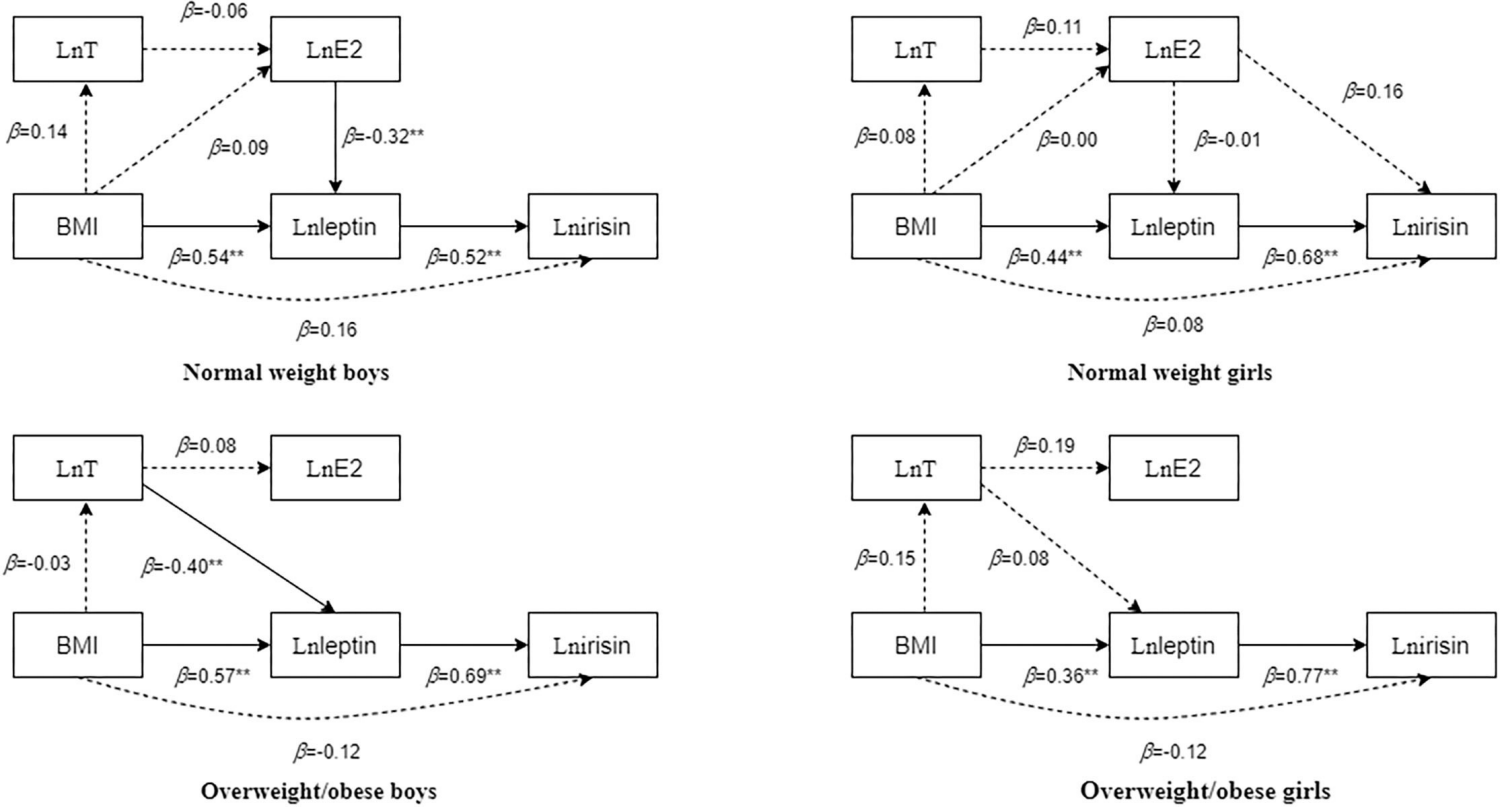
Multifactor regression results of body mass index (BMI), testosterone (T), oestradiol (E2), leptin, and irisin in children and adolescents with different body types after adjusting for age. The dotted line in the figure indicates that the regression result is not statistically significant, whereas the solid line indicates that the regression result is statistically significant. The numbers beside the arrows show the standardized regression coefficients; ^**^p < 0.01.

Multiple linear regression analysis was conducted with irisin, leptin, E2, and T as dependent variables. Figure 1 shows that leptin was an independent predictor of irisin, and that BMI was an independent predictor of leptin in all boys and girls. In normal-weight boys, E2 was an independent predictor of leptin. In overweight/obese boys, T was an independent predictor of leptin.

### Path Analysis Results of BMI, T, E2, Leptin, and Irisin in Children and Adolescents

**Figure 2:**
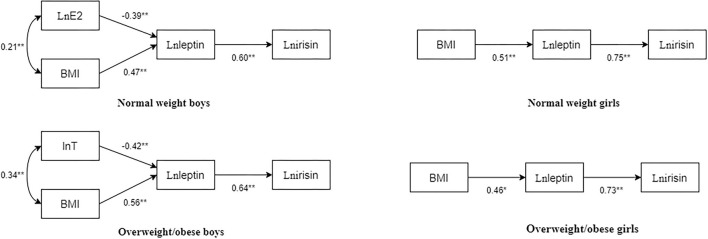
Path diagram of BMI, T, E2, leptin, and irisin in children and adolescents with different body types. The numbers beside the arrows show the standardized path coefficients; *p < 0.05; **p < 0.01.

Based on the correlation and regression results, path structural equation models of BMI, T, E2, leptin, and irisin in children and adolescents with different body types were constructed (Figure 2).

In normal-weight boys, the fit indices of the model were as follows: χ^2^/df = 0.845, normed fit index (NFI) = 0.982, incremental fit index (IFI) = 1.003, Tucker-Lewis index (TLI) = 1.011, comparative fit index (CFI) = 1, goodness-of-fit index (GFI) = 0.992, stangardized root mean square residual (SRMR) = 0.03, *p* = 0.430. Particularly, E2 had a significant negative predictive effect on leptin (standardized path coefficient β = −0.39, *p* < 0.01), BMI had a significant positive predictive effect on leptin (standardized path coefficient β = 0.47, *p* < 0.01), and leptin significantly and positively predicted irisin (standardized path coefficient β = 0.60, *p* < 0.01).

In overweight/obese boys, the fit indices of the model were χ^2^/df = 2.562, NFI = 0.956, IFI = 0.973, TLI = 0.915, CFI = 0.972, GFI = 0.977, SRMR = 0.04, and *p* = 0.077. Particularly, T had a significant negative predictive effect on leptin (standardized path coefficient β = −0.42, *p* < 0.01), BMI had a significant positive predictive effect on leptin (standardized path coefficient β = 0.56, *p* < 0.01), and leptin significantly and positively predicted irisin (standardized path coefficient β = 0.64, *p* < 0.01).

In normal-weight girls, the fit indices of the model were χ^2^/df = 1.545, NFI = 0.982, IFI = 0.994, TLI = 0.981, CFI = 0.994, GFI = 0.987, SRMR = 0.03, *p* = 0.214. Particularly, BMI had a significant positive predictive effect on leptin (standardized path coefficient β = 0.51, *p* < 0.01), and leptin positively predicted irisin (standardized path coefficient β = 0.75, *p* < 0.01).

In overweight/obese girls, the fit indices of the model were χ^2^/df = 1.316, NFI = 0.983, IFI = 0.996, TLI = 0.987, CFI = 0.996, GFI = 0.989, SRMR = 0.03, and *p* = 0.251. Particularly, BMI had a significant positive predictive effect on leptin (standardized path coefficient β = 0.46, *p* < 0.01), and leptin significantly positively predicted irisin (standardized path coefficient β = 0.73, *p* < 0.01).

### Indirect Effect Test

The bootstrap method with bias correction percentile was used to repeatedly sample 5,000 times to test the above paths. The results in Table 3 showed that the above paths have significant indirect effects. In normal-weight boys, the indirect paths from E2 to irisin and the indirect paths from BMI to irisin were statistically significant [standardized indirect effect coefficient: −0.24 (*p* < 0.01) and 0.29 (*p* < 0.01), respectively]. In overweight/obese boys, the indirect paths from T to irisin and the indirect paths from BMI to irisin were statistically significant [standardized indirect effect coefficient: −0.27 (*p* < 0.01) and 0.36 (*p* < 0.01), respectively]. In normal-weight girls, the indirect paths from BMI to irisin were statistically significant [standardized indirect effect coefficient: 0.38 (*p* < 0.01)]. In overweight/obese girls, the indirect paths from BMI to irisin were statistically significant [standardized indirect effect coefficient: = 0.34 (*p* < 0.01)].

**Table 3:** Bootstrap analysis of significance test of indirect effect.

**Pathway**	**β**	**SE**	***P***	**95%CI**
**Normal-weighted boys**				
LnE2 → Lnleptin → Lnirisin	−0.24	0.04	<0.01	−0.32~-0.16
BMI → Lnleptin → Lnirisin	0.29	0.08	<0.01	0.14~0.44
**Overweight/obese boys**				
LnT → Lnleptin → Lnirisin	−0.27	0.06	<0.01	−0.37~0.15
BMI → Lnleptin → Lnirisin	0.36	0.05	<0.01	0.26~0.46
**Normal-weighted girls**				
BMI → Lnleptin → Lnirisin	0.38	0.06	<0.01	0.27~0.51
**Overweight/obese girls**				
BMI → Lnleptin → Lnirisin	0.34	0.14	<0.05	0.05~0.58

## Discussion

In recent decades, obesity in children and adolescents has become a serious health problem worldwide, leading to lifelong clinical and psychosocial consequences and other comorbidities, such as impaired glucose tolerance (31) and abnormalities associated with growth and puberty (32). The relationships between adipokines in pediatric obesity are still not very clear. Our study indicated that serum leptin and irisin concentrations in girls were significantly higher than those in boys. Leptin and irisin concentrations in overweight/obese children and adolescents were significantly higher than those in normal-weight children and adolescents. Serum leptin levels in children and adolescents play a mediating role in the relationship between BMI and irisin. The E2 was negatively correlated with leptin in normal-weight boys, whereas T was negatively correlated with leptin in overweight/obese boys.

Leptin levels have been shown to exponentially increase with adipose tissue mass (33), while increased leptin levels in obese people can directly downregulate leptin receptor levels or block post-receptor signal transduction, leading to leptin resistance and promoting leptin secretion, which causes hyperleptinemia. Therefore, obesity in humans may represent a state of leptin resistance (34), which can cause IR through a variety of pathways. Hence, leptin is a recognized indicator of IR (35). Stengel et al. (11) found that irisin was positively correlated with body fat percentage and BMI with abdominal obesity. However, some studies have shown that the serum irisin level of overweight and obese people is lower than that of normal-weight people and that irisin is negatively correlated with overweight and obesity (5), but other studies have shown no clear link (36, 37). Therefore, the relationship between BMI and serum irisin concentration is highly controversial. The total concentration of irisin is higher in obese people at rest than in lean people, suggesting that the elevated levels of irisin in obese people are chronically elevated due to changes in metabolic signals associated with obesity (29).

This study showed that there was sexual dimorphism in serum leptin concentration in normal-weight children and adolescents, with girls having a higher concentration than boys, consistent with the results of relevant studies (38, 39). The higher levels of leptin in girls are attributed to the higher fat content in girls than in boys. In addition, sex hormones may play a role in leptin synthesis and/or release. Irisin also has sexual dimorphism in both normal-weight and overweight/obese children and adolescents, with girls' irisin levels higher than boys'. One study found that resting girls have higher levels of irisin than boys (40). Irisin is a factor acting in and secreted by muscles and fat. Hence, sexual dimorphism of irisin may be related to the different distribution of skeletal muscle, fat, and other body components in boys and girls (41).

Through path analysis, this study found that BMI was a positive and significant predictor of leptin in children and adolescents with different body types. The BMI was not a direct predictor of irisin, but leptin had a certain mediating effect on the relationship between BMI and irisin. Studies have confirmed that leptin concentration in obese people is significantly increased, leptin concentration is positively correlated with IR, irisin concentration is positively correlated with IR (42), and leptin regulates FNDC5 expression and/or irisin production (43, 44). Therefore, the association between BMI and irisin concentration may be due to leptin mediation. In normal-weight boys, in addition to the BM → leptin → irisin pathway, there is also a negative E2 → leptin → irisin pathway, wherein E2 is negatively correlated with leptin different from the results of Lou et al. (45). This may be because E2 in boys is mainly transformed from T through aromatization. Hence, boys of normal weight have a higher T level and a corresponding increase in E2 level. Therefore, the inhibition of leptin secretion is not reflected in the T level but in the E2 level. In overweight/obese boys, in addition to the BMI → leptin → irisin pathway, there was also a negative T → leptin → irisin pathway, in which T was negatively correlated with leptin. Existing studies have also confirmed that T inhibits the secretion of leptin, and serum irisin is negatively correlated with T (46, 47). *In vitro* studies of cultured human adipocytes also revealed the inhibitory effect of T and dihydrotestosterone (DHT) on leptin. The serum T levels of obese boys decreased, and the inhibitory effect on leptin was weakened, leading to a significant increase in leptin level (48). Moreover, there was no significant inhibitory effect of E2/T on leptin in normal-weight and overweight/obese girls. Li et al. (49) found that there was no correlation between serum leptin levels, E2, and T in girls during pubertal development. In addition, compared with boys, girls had lower T levels, which may not be enough to inhibit leptin. Existing studies have reported an inconsistent relationship between E2 and leptin in women. A large number of studies have found a positive (50, 51) or negative correlation (52), while some have found no correlation between E2 and leptin (53, 54), which seems to be a complicated issue. The path association may be related to genetic and epigenetic factors. Studies reported that the expression level of fat mass and obesity associated gene (FTO) and pleomorphic adenoma gene 1 (PLAG1) genes associated with obesity was significantly related to the concentration of leptin (55, 56).

This study found that there may be a negative feedback effect of sex hormones on leptin and irisin in boys. Leptin and irisin are thought to play a role in the connection between body fat storage and the hypothalamic-pituitary-gonad (HPG). Leptin controls the normal physiology of the reproductive system and interacts with the HPG axis through a complex mechanism that links energy homeostasis to reproduction (16), while irisin stimulates gonadotropin gene expression in pituitary cells (57). This may indicate that the relationship between leptin, irisin, and sex hormones is governed by a complex feedback loop, rather than a simple one-way relationship. The existing data showed that irisin has positive effects on pituitary functions as chronic exposure to irisin produced significant increases in serum LH and T levels in male rats (26). *In vivo*, irisin could promote the secretion of FSH and LH in female rats (58). In addition, *in vitro*, irisin treatment increased the expression of FSH and LH in the pituitary cells by improving the stability of transcription. Hence, irisin may play a GnRH-like role in the HPG axis (57). Therefore, irisin levels in circulation change significantly during adolescence, pregnancy, and postpartum when the functions of the HPG axis are active. However, the mechanism by which leptin and irisin into with sex hormones are largely unknown.

There are some limitations in the study that should be noted. First, this is a cross-sectional study, and it cannot explain the causal relationship between hormones. Second, the body shape of children and adolescents can be influenced by other confounding factors, such as diet and exercise, which this study did not take into consideration. Third, the research population only included Chinese children and adolescents, which prevents explanation of the situation of children and adolescents in other countries. The research results need to be verified by further studies.

## Conclusion

This study indicated that the leptin and irisin concentrations of overweight/obese boys and girls were significantly higher than those with normal weight. In addition, there was sexual dimorphism in serum irisin concentration in both normal or overweight/obese children and adolescents, with girls having a higher concentration than boys. The indirect effects of BMI on irisin *via* leptin exist in children and adolescents of different body types both in boys and girls. The E2 was negatively correlated with leptin in normal-weight boys, whereas T was negatively correlated with leptin in overweight/obese boys.

## Data Availability Statement

The original contributions presented in the study are included in the article/supplementary files, further inquiries can be directed to the corresponding author/s.

## Ethics Statement

The studies involving human participants were reviewed and approved by Medical Ethics Committee of the Bengbu Medical College ([2015] No. 003). Written informed consent to participate in this study was provided by the participants' legal guardian/next of kin.

## Author Contributions

LZ conceptualized and designed the study, analyzed and interpreted the data, drafted the initial manuscript, and reviewed and revised the manuscript. XP, YW, RC, and ZZ collected data and revised the manuscript. LF conceptualized and designed the study, coordinated and supervised data collection, and critically reviewed the manuscript for important intellectual content. All authors contributed to the study conception and design and read and approved the final manuscript.

## Funding

This project was supported by grants from the National Natural Science Foundation of China (81502823), 512 Talent Cultivation Plan of Bengbu Medical College (by51201204), and the Scientific Research and Innovation Team Project of Bengbu Medical College (BYKC201901).

## Conflict of Interest

The authors declare that the research was conducted in the absence of any commercial or financial relationships that could be construed as a potential conflict of interest.

## Publisher's Note

All claims expressed in this article are solely those of the authors and do not necessarily represent those of their affiliated organizations, or those of the publisher, the editors and the reviewers. Any product that may be evaluated in this article, or claim that may be made by its manufacturer, is not guaranteed or endorsed by the publisher.
